# Shifting from a thermal-constrained to water-constrained ecosystem over the Tibetan Plateau

**DOI:** 10.3389/fpls.2023.1125288

**Published:** 2023-04-19

**Authors:** Chaoyi Xu, Dan Liu, Xiaoyi Wang, Tao Wang

**Affiliations:** ^1^ State Key Laboratory of Tibetan Plateau Earth System, Resources and Environment (TPESRE), Institute of Tibetan Plateau Research, Chinese Academy of Sciences, Beijing, China; ^2^ University of Chinese Academy of Sciences, Beijing, China

**Keywords:** date of peak vegetation growth, climate constraint, alpine grassland, Tibetan Plateau, ecosystem production

## Abstract

**Introduction:**

Understanding the seasonality of vegetation growth is important for maintaining sustainable development of grassland livestock systems over the Tibetan Plateau (TP). Current knowledge of changes in the seasonality of TP grasslands is restricted to spring and autumn phenology, with little known about the date of peak vegetation growth, the most relevant quantity for grassland productivity.

**Methods:**

We investigate the shifts of the date of peak vegetation growth and its climatic controls for the alpine grasslands over the TP during 2001–2020 using a framework based on the law of minimum, which is based on the assumption that peak vegetation growth would be consistent with the peak timing of the most limiting climatic resource.

**Results:**

The date of peak vegetation growth over the TP advanced by 0.81 days decade-1 during 2001–2020. This spring-ward shift mainly occurs in the semi-humid eastern TP, where the peak growth date tracks the advancing peak precipitation, and shifted towards the timing of peak temperature. The advancing peak growth over the eastern TP significantly stimulated the ecosystem production by 1.99 gCm-2 year-1 day-1 during 2001–2020, while this positive effect weakened from 3.02 gCm-2 year-1 day-1 during 2000s to 1.25 gCm-2 year-1 day-1 during 2010s.

**Discussion:**

Our results highlighted the importance of water availability in vegetation growth over the TP, and indicated that the TP grassland is moving towards a tipping point of transition from thermal-constrained to water-constrained ecosystem under the rapid warming climate.

## 1 Introduction

For decades, researchers have been interested in the seasonal timing of spring arrival and autumn senescence in the Northern Hemisphere, since variations in the timing of these events can have profound influences on shaping the net carbon balance over cold northern ecosystems (Keeling et al., 1996; Cleland et al., 2007; Piao et al., 2008) and on feedbacks to the climate by changing the surface albedo and evapotranspiration (Bonan, 2008; Peñuelas et al., 2009). In contrast, the date of peak vegetation growth, which corresponds to the period when climatic constraints on plant growth are the least, and generally coincides with the timing of maximum ecosystem carbon uptake (Park et al., 2019), has received much less attention. However, evidence is emerging that changes in the peak growth date could significantly impact terrestrial ecosystem productivity (Buitenwerf et al., 2015; Gonsamo et al., 2018) and seasonal (peak-to-trough) amplitude of atmospheric CO_2_ in northern high latitudes (Gonsamo et al., 2018). It is imperative to develop a robust understanding of the peak growth date and its long-term changes in a warming atmosphere, to complete our knowledge on the interaction between climate and the seasonality of vegetation activity that is currently predominantly restricted to spring and autumn phenology.

The Tibetan Plateau (TP), with a mean elevation of more than 4000 m, harbors the largest area of alpine grasslands outside the polar regions (Chen et al., 2014a). In addition to their role in the carbon cycle (Piao et al., 2009), TP alpine ecosystems also play an important role in slowing down the rate of local warming (Shen et al., 2015) and in changing the spatial pattern of east Asian summer monsoon precipitation, by modifying the strength of TP as a heat source or sink (Zuo et al., 2011). Revealing the peak growth date and its dynamics, will not only complete the picture of ecosystem greenness changes, but is also conducive to understanding the changing temperature seasonality and the plateau-induced changes in east Asia’s summer precipitation patterns. Currently, we lack such a description of the spatial-temporal patterns of the peak growth date and its climatic controls over the Tibetan Plateau.

Alpine ecosystems on the TP are widely considered to be thermal-constrained (Liu et al., 2018), an assumption which has been given further support by Huang et al. (2019) who showed that the optimum ecosystem-level photosynthetic temperature is well below current growing-season daily maximum temperature. This observation led us to hypothesize that the date of peak vegetation growth would occur earlier, similar to the response of the peak growth date of Arctic and boreal ecosystem to warming (Park et al., 2019). On the other hand, however, most of the alpine ecosystems on the TP are characterized by a semi-arid and semi-humid climate (Yang et al., 2014), and there is a growing recognition that Tibetan plant growth is also regulated by the availability of moisture (Shen et al., 2015; Li et al., 2016; Ding et al., 2018; Liu et al., 2019). For example, Li et al. (2016) used *in-situ* species phenological observations to show that the timing of leaf unfolding over alpine meadows was synchronized with the onset of Indian monsoon precipitation. Furthermore, in contrast to other thermal-constrained ecosystems such as those in the Arctic, the TP generally receives more solar insolation, which could allow higher evapotranspiration and consequently leave less soil water available for plant growth (Yao et al., 2008). However, these studies do not demonstrate unequivocally which climatic factor constrains the vegetation growth, or how it changes in response to the observed rapid warming and nonhomogeneous precipitation changes.

In this study, we analyzed the climatic constraints of vegetation growth over the TP using the framework proposed by Park et al. (2019). We mapped the date of peak vegetation growth using continuous satellite observations from 2001 to 2020 over the TP, and investigated the spatial-temporal pattern of the primary climate constrains for vegetation growth over the Tibetan Plateau.

## 2 Materials and methods

### 2.1 Study regions

The TP is the largest alpine ecosystem in the world and has an extremely cold climate. The growing season temperature over the TP is about 12°C, which is similar to that in the Arctic (Huang et al., 2019). In contrast to the Arctic where the radiation is weak (~105 W m^-2^) (Hamman et al., 2016), the TP receives strong solar radiation (~204 W m^-2^), which is much stronger than that in the surrounding areas based on the observations from the meteorological stations of China Meteorological Administration (Yang et al., 2014). The precipitation regime over the TP is mainly shaped by the interaction between the Indian summer monsoon and the mid-latitude westerlies (Yao et al., 2012). The annual precipitation on the TP decreases from over 1000 mm in the southeast to less than 50 mm in the northwest (Kuang and Jiao, 2016), and the aridity status changes from humid and semi-humid in the southeast, to semi-arid in the central plateau, and arid in the northwest. Nearly 59.3% of the TP (1.5×10^6^ km^2^) is covered by alpine grasslands (Zhang et al., 2014). The grassland ecosystems vary along the aridity gradient from alpine meadow (0.7×10^6^ km^2^) in the southeast, to alpine steppe (0.58×10^6^ km^2^) in the northwest (Zhang et al., 2014).

### 2.2 Analyzing framework

**Figure 1:**
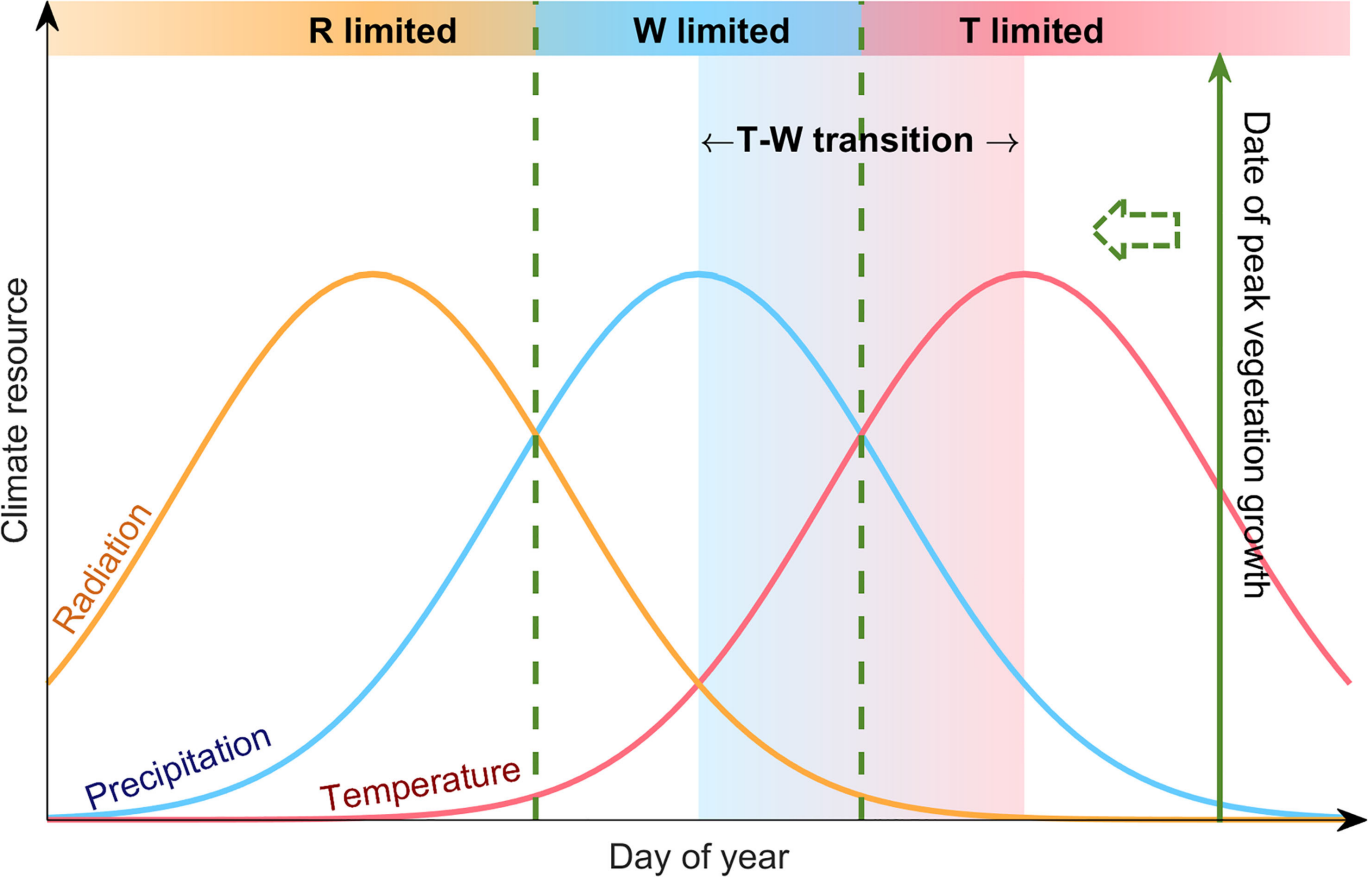
Conceptual illustration of the analyzing framework based on “law of minimum”. The curves show the standardized seasonal cycle of radiation (orange), precipitation (blue) and temperature (red) over the Tibetan Plateau. The solid green line shows the date of peak vegetation growth. The shadings mark different cases for climate constrains on vegetation growth. For instance, when the date of peak vegetation growth occurs around the temperature peak, it means that the most abundant water availability did not lead to vegetation growth reaching its peak, until adequate heat supports peak vegetation growth, indicating that the ecosystem is thermal-limited (T-limited). Similarly, an ecosystem with the date of peak vegetation growth close to the precipitation peak would be a water-limited ecosystem (W-limited), and we define the case when the date of peak vegetation growth occurs between the precipitation and temperature peaks as a transition state between thermal- and water-limited ecosystem (T-W transition).

Our analysis was conducted based on the framework proposed by Park et al. (2019). This framework analyzes climatic constrains for plant growth based on the law of minimum. It relays on two fundamental principles: (1) vegetation growth would be seasonally consistent with radiation if the ecosystem is not constrained by other climatic resources (e.g. temperature as a proxy for thermal energy, and precipitation as a proxy for water availability). Under this condition, the timing of peak vegetation growth would be close to the peak radiation. (2) When the ecosystem is constrained by thermal or hydrological climatic resources, the timing of peak vegetation growth would be close to the peak timing of the most limiting factor. This framework has been proven to be reliable, and was applied to investigate the climatic constraints for the peak photosynthesis over the northern hemisphere (Park et al., 2019; Zhao et al., 2022). Over the Tibetan Plateau, the timing of peak radiation occurs much earlier than the temperature and precipitation peaks. Due to the high elevation, the alpine ecosystem over the TP receives much stronger solar radiation than other temperate regions (Chen et al., 2014b), and radiation is not likely to be the limiting climatic resource for vegetation growth. Therefore, in this study we mainly focus on thermal and water constraints for peak vegetation growth. We also define a scenario when peak vegetation growth occurs between the peak temperature and peak precipitation as a transition state between thermal- and water-limited ecosystem (**Figure 1**).

### 2.3 Satellite-based vegetation indices and gross primary productivity

We used the normalized difference vegetation index (NDVI) to detect the peak growth date. The NDVI was derived from the Nadir Bidirectional Reflectance Distribution Function-Adjusted Reflectance (NBAR) from the MODerate-resolution Imaging Spectroradiometer (MODIS) (MCD43C4 v061). This latest version of the MODIS product does not have the major defect of sensor degradation that was present in the early datasets (Hilker et al., 2014) and should, therefore, be able to provide a more accurate estimate of changes in peak growth over the TP. The MCD43C4 dataset provides the reflectance for seven bands at daily time scale. We calculated NDVI from the reflectance (*ρ*) in the red and NIR bands as (*ρ*NIR - *ρ*Red)/(*ρ*NIR + *ρ*Red). We only used data flagged as “good quality”. The derived NDVI dataset has a spatial resolution of 0.05° × 0.05°, and cover the period from 2001 to 2020. We interpolated it into a spatial resolution of 0.1° × 0.1° for further analysis. We only considered vegetated regions, and pixels with annual NDVI below 0.1 was excluded as bare ground.

To test the robustness of our analysis, we also included four alternative vegetation indices and gross primary production (GPP) products to detect the peak growth date, including the NDVI derived from MODIS surface reflectance products (MOD09A1, v061), the GOSIF GPP product (Li and Xiao, 2019), the CSIF product (Zhang et al., 2018), and the PML-V2 GPP product (Zhang et al., 2019). We calculated NDVI from the MOD09A1 product for the period of 2001–2020, with a spatial resolution of 500 m and 8-day time step. We only used data with good quality, and records marked as cloud, cloud shadow, and aerosols were removed from analysis. GOSIF-GPP is the fine-resolution dataset developed from the OCO-2 based Global SIF product (GOSIF) and the SIF-GPP linear relationship at a 0.05° spatial resolution and 8-day time step (Li and Xiao, 2019). This GPP dataset covers the period 2001–2020. CSIF is generated by training artificial neural networks with MODIS surface reflectance and OCO-2-based solar induced chlorophyll fluorescence, with a 4-day temporal resolution and spatial resolution of 0.05° × 0.05° (Zhang et al., 2018). In this study, we used clear-sky instantaneous CSIF for the period 2001–2019. The PML-V2 dataset estimates GPP and ET simultaneously at 500m resolution and daily time scale using a coupled diagnostic biophysical model (PML-V2) forced by MODIS derived leaf area index, albedo, emissivity, and GLDAS meteorological datasets (Zhang et al., 2019). It was calibrated using 26 eddy flux (EC) stations in China. All these datasets were resampled to a spatial resolution of 0.1° × 0.1° and interpolated to a daily time step using spline interpolation at each pixel. We further used the PML-V2 GPP data to investigate the influence of the shift of peak growth date on ecosystem productivity.

### 2.4 Climate dataset

The climate variables, including near-surface air temperature, precipitation, and incident solar radiation were derived from the reanalysis product of Indian Monsoon Data Assimilation and Analysis reanalysis (IMDAA) project. The IMDAA product is a regional reanalysis product that assimilates a variety of conventional observations including satellite retrievals and synthetic data though the Met Office Unified Model (UM) using four-dimensional variational (4D-Var) data assimilation method (Mahmood et al., 2018). It provides consistent dataset of high-resolution fields (0.12° × 0.12°) from 1979–2020 (Rani et al., 2021). We integrated the hourly dataset to a daily time step and interpolated to a spatial resolution of 0.1° × 0.1°.

We also analyzed *in situ* soil moisture from nine sites (**Table 1**) to investigate the influence of permafrost thaw on the water availability for plant growth during peak season. These sites are located along the Qingzang railway, and the data was obtained from Zhao et al. (2021). We used the soil moisture observation at top layer of 10cm depth. For sites that lack observations at 10cm depth, we used records from the adjacent layer of 5cm or 15cm.

**Table 1:** List of sample sites of soil moisture observations used in this study.

Name	Location	Period
Ch04	31.82°N, 91.74°E	2001–2006
Ch06	35.62°N, 94.06°E	2005–2016
QT01	35.14°N, 93.04°E	2004–2014
QT03	34.82°N, 92.92°E	2004–2014
QT04	33.07°N, 91.94°E	2006–2012
QT05	33.96°N, 92.34°E	2004–2008
QT08	35.22°N, 93.08°E	2012–2018
QT09	35.72°N, 94.13°E	2011–2018
ZNHAL	35.49°N, 91.96°E	2014–2020

### 2.5 Detection of peak dates

To calculate the peak vegetation growth date, we applied the singular spectrum analysis (SSA) to the daily NDVI or GPP within each year at each pixel, so as to remove high frequency noise and retain the relatively low frequency signals such as the seasonal cycle (Keenan et al., 2014; Zhou et al., 2017; Park et al., 2019). The peak growth date is then calculated as the date on which the filtered NDVI value reaches its annual maximum. The same method is applied to daily climatic variables (precipitation, temperature, and surface downward short-wave radiation) to identify the peak dates.

## 3 Results

### 3.1 The spatial pattern of the date of peak vegetation growth

**Figure 2:**
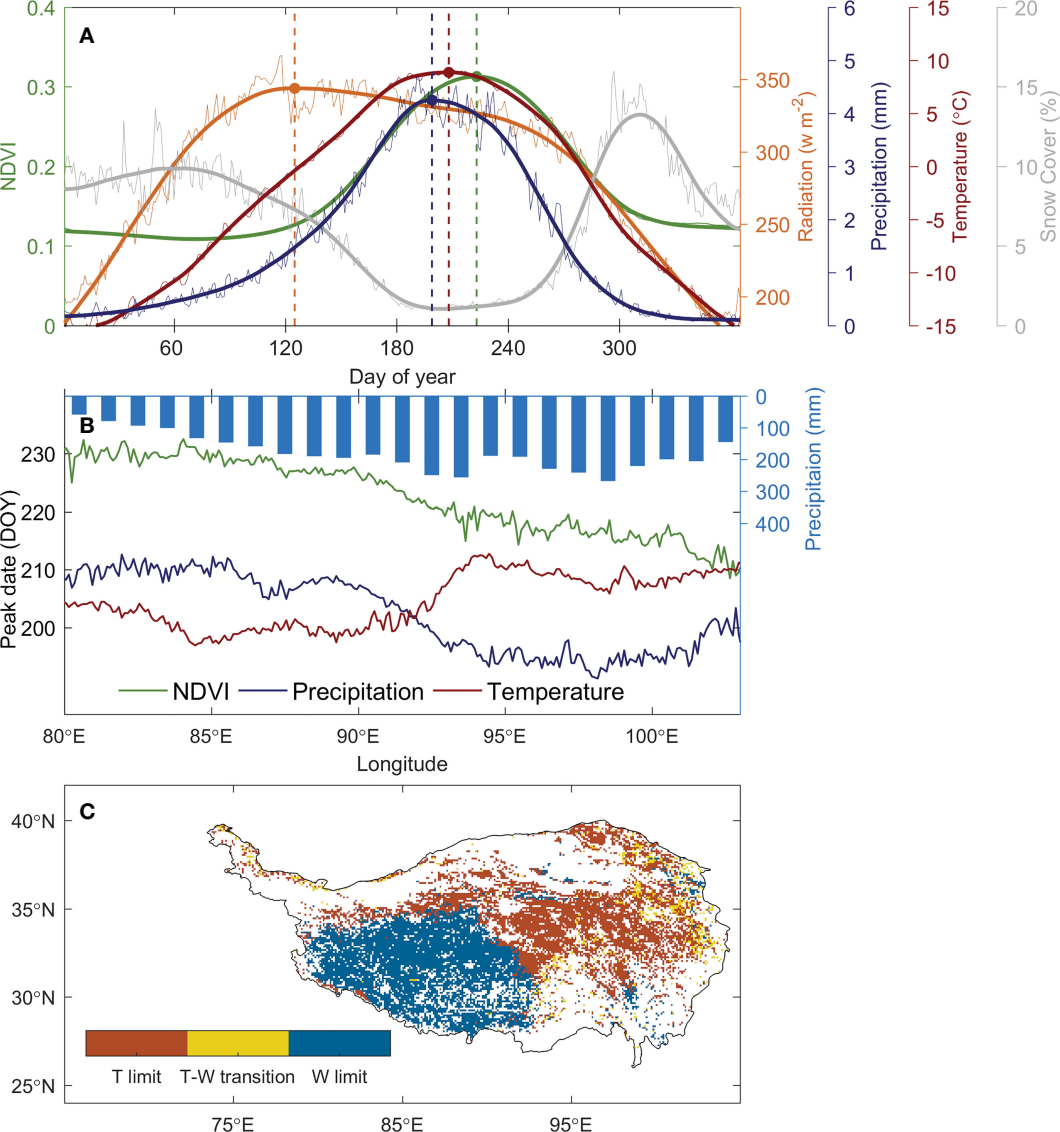
The climate constraints for the alpine grasslands over the TP and its spatial distribution. **(A)** shows the seasonal cycle of solar radiation (orange line), precipitation (blue line), temperature (red line), NDVI (green line), and snow cover (grey line). The dots and vertical dash lines mark the timing of the peaks. **(B)** illustrates the relative positioning of the timing of temperature (red) and precipitation (blue) peaks against NDVI peaks (green) along the longitude. The blue bars show the precipitation gradient along longitude over the TP. **(C)** shows the spatial pattern of climate constraints over the TP under the definition in **Figure 1** based on the law of minimum.

The date of peak vegetation growth over the TP occurs at early August (day of the year, DOY 213) (**Figure 2A**). It is months later than the date of peak radiation (DOY 125), and is close to the date of peak temperature (DOY 208) and peak precipitation (DOY 199) (**Figure 2A**), suggesting that the date of peak vegetation growth occurs around the period when the thermal and hydrological condition is most suitable for vegetation growth. During this period, the snow cover over the TP reaches its minimum (**Figure 2A**) and the top-layer soil is thawed due to the warm climate in summer (Wu and Zhang, 2010). This multi-year averaged peak growth date suggests a long-term acclimation of vegetation growth to the regional climate over the TP, and it supports our assumption that the date of peak growth occurs around the peak of most constraining factor.

Spatially, the date of peak vegetation growth delays from late-July in the east (DOY 215 ± 5) to mid-August (DOY 228 ± 2) in the west along the aridity gradient (**Figure 2B**). In the humid and semi-humid region where the annual precipitation is reaches 250 mm year^-1^, the peak precipitation (DOY 197) occurs 11 ± 5 days before the peak temperature (DOY 208), and the vegetation growth reaches the peak at 10 ± 7 days later than the date of peak temperature, suggesting that the alpine meadow in the humid and semi-humid region is constrained by temperature. In the semi-arid region with an annual precipitation below 200 mm year^-1^, the peak precipitation lags behind peak temperature for 8 ± 3 days, and the vegetation growth peaks at 20 ± 2 days later than the peak precipitation. This indicates that the water availability is the primary constrain for the ecosystem in this semi-arid region. Spatially, 46% of the region is thermal-constrained, with 77% of which located in the semi-humid east, and 48% of the region is water-constrained, with 85% of which lay in the semi-arid west. For 6% of the region, the timing of peak vegetation growth occurs between the peak temperature and peak precipitation, suggesting it in a transition state of thermal- and water-constrained ecosystem (**Figure 2C**). This spatial pattern of peak growth date and the relative positioning against the climatic peaks demonstrated that our analysis framework well represents the primary constrains of vegetation growth over the plateau.

### 3.2 Shifting date of peak vegetation growth and its relative positioning against climatic peaks

**Figure 3:**
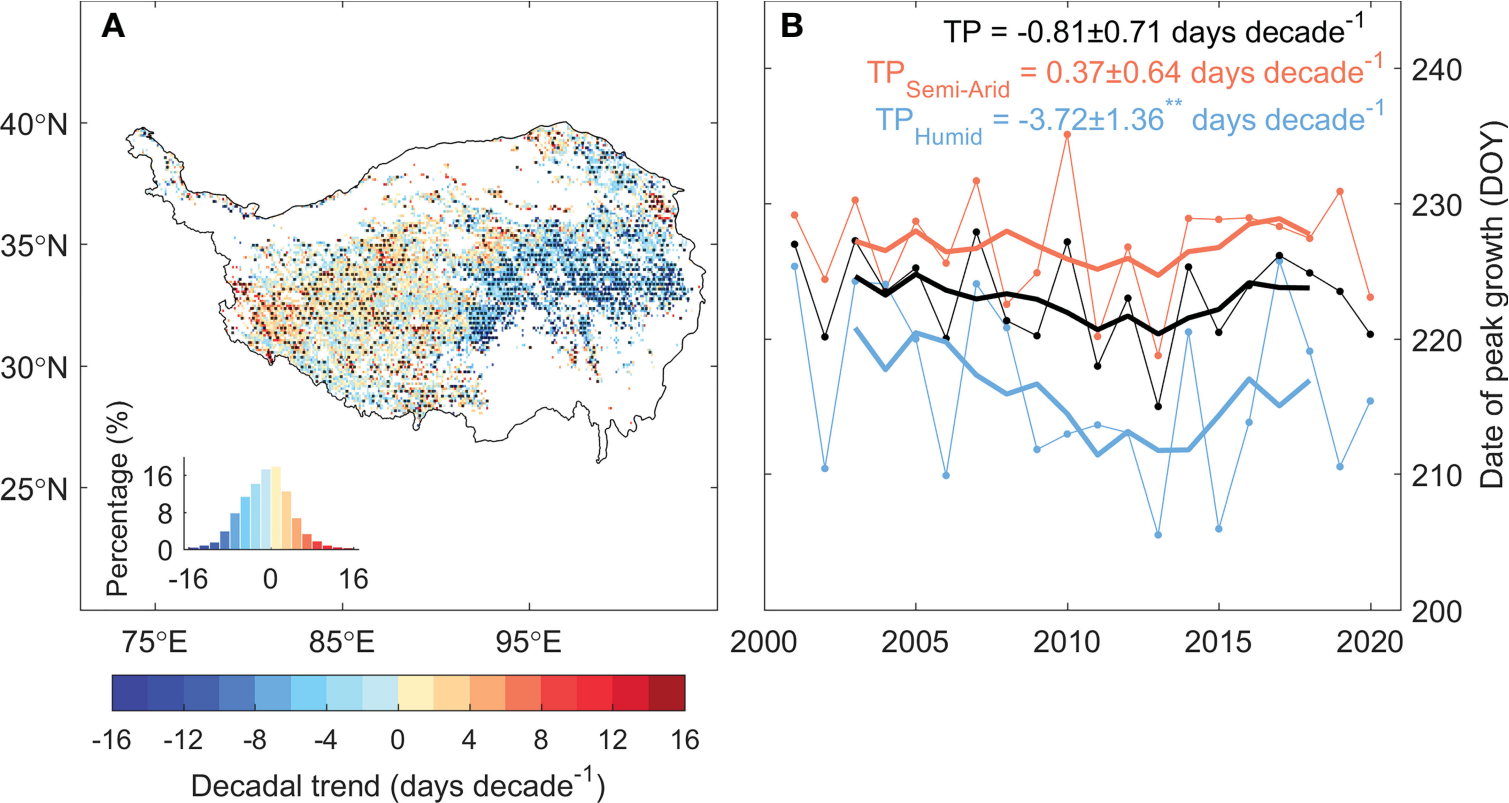
The temporal trend of the date of peak vegetation growth for the period 2001–2020. **(A)** shows the spatial pattern of the trend of the date of peak vegetation growth, and **(B)** shows the time series of the date of peak vegetation growth for the TP (black), semi-arid western TP (orange) and semi-humid eastern TP (blue). Values marked with ** suggest that the trend is significant at the level of *P* < 0.05. Black dots in the spatial pattern denote that the trend is significant at the level of *P* < 0.05.

The date of peak vegetation growth over the TP shifted notably during 2001–2020, while the shifting patterns are divergent between the eastern and western plateau (**Figure 3A**). In the east, 58% of the grasslands showed significant advancing peak growth. On average, the peak growth date of eastern plateau significantly advanced at a rate of 3.72 ± 1.36 days decade^-1^, which keeps in pace with the advancing date of the onset of growing season (Shen et al., 2022). In the west, however, the peak vegetation growth delayed at a rate of 0.37 ± 0.64 days decade^-1^, with 20% of the region showed a significant delaying trend. On average, the date of peak vegetation growth over the TP advances at a rate of 0.81 ± 0.71 days decade^-1^ during 2001–2020 (**Figure 3B**). Results from other four vegetation indices and GPP products showed similar shifting patterns, demonstrated the robustness of the changes in peak growth dates over the TP (**Supplementary Figure 1**).

**Figure 4:**
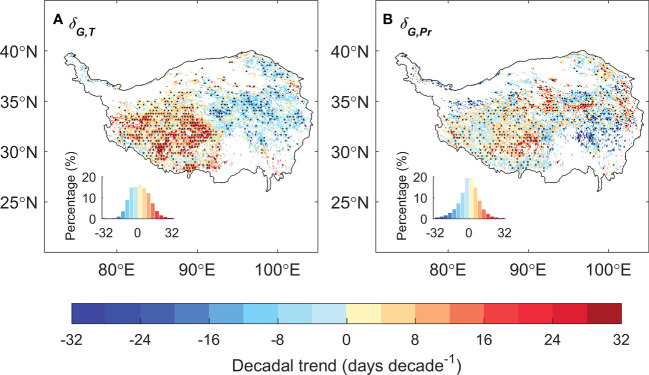
The spatial pattern of the decadal trend for the difference of the date of peak vegetation growth against the date of peak temperature (*δ_G,T_*, **A**) and peak precipitation (*δ_G,Pr_*, **B**).

**Figure 5:**
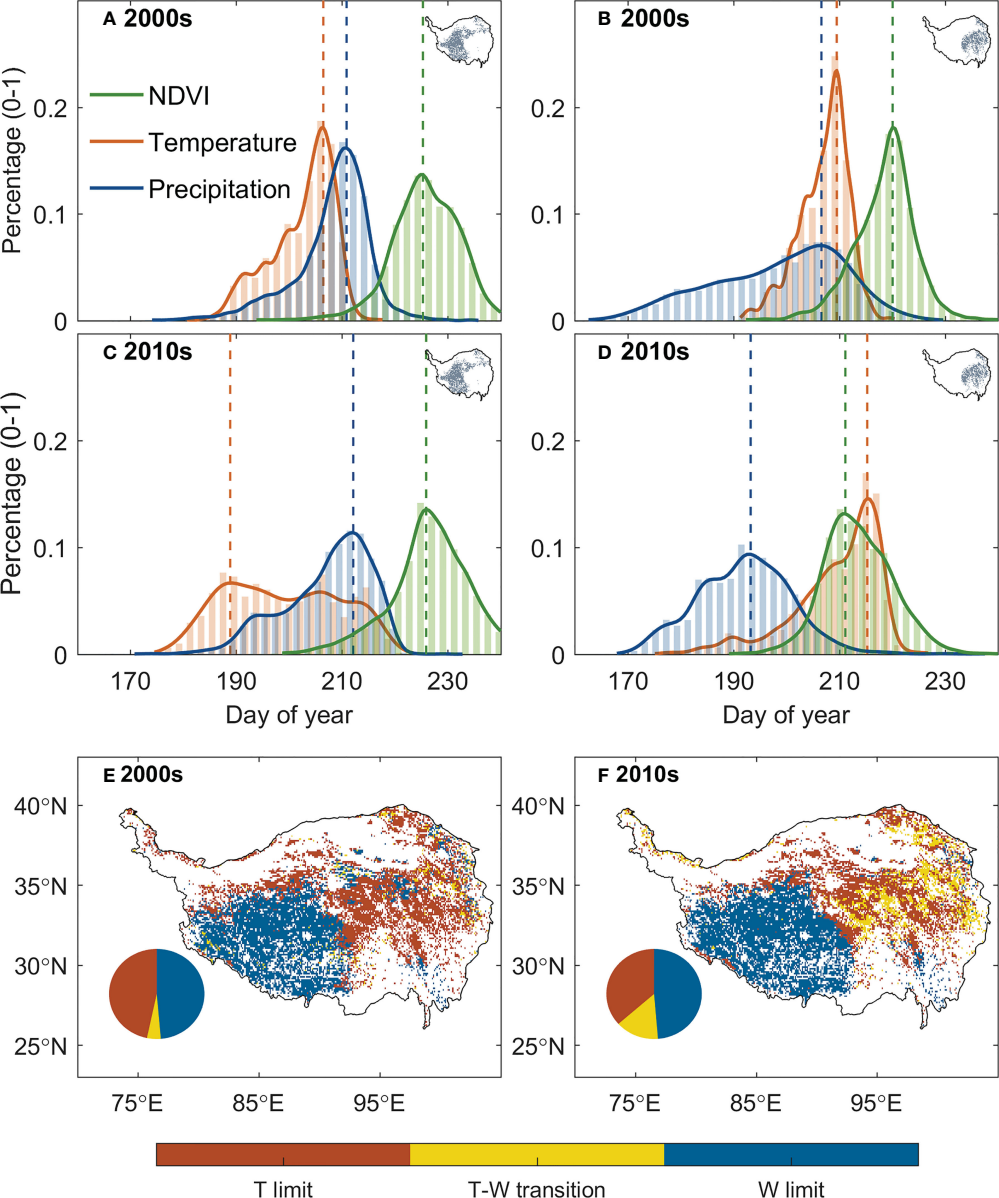
Changes in the climate constraints between 2000s (2001–2010) and 2010s (2011–2020). **(A–D)** show the statistical distribution of the date of peak vegetation growth (green), peak temperature (red), and peak precipitation (blue) for the semi-arid western TP and semi-humid eastern TP for the two periods. The decadal average of the date of the peaks for each pixel was used to generate the histogram, and the inset maps with grey dots show the spatial domain of the histogram. **(E, F)** illustrate the spatial distribution of the climate constraints over the TP for the two periods. The pie plots show the percentage of the regions that are thermal-limited (red), water-limited (blue) or under transition state between thermal- and water-limited ecosystem (yellow).

We then analyzed the changes of the relative positioning between the date of peak vegetation growth and that of climate peaks. The relative timing between the dates of peak vegetation growth and peak temperature (*δ_G,T_*) has changed significantly over the past 20 years, with significant increasing trend in the east and decreasing trend in the west, while that against the date of peak precipitation (*δ_G,Pr_*) is much less notable (**Figure 4**). We then specifically analyzed the relative positioning of peak vegetation growth and climate peaks for 2000s (2001–2010) and 2010s (2011–2020) for the eastern and western plateau respectively (**Figures 5A–D**). In the eastern plateau, the climate shifts as an “advancing peak precipitation – delaying peak temperature” pattern. The date of peak precipitation advanced by 6 days from 198 ± 12 in 2000s to 192 ± 5 in 2010s, and the date of peak temperature varies delays from 207 ± 5 in 2000s to 210 ± 8 in 2010s. In response to these shifts in climate, the date of peak vegetation growth tracks the advancing peak precipitation and shifted spring-ward for 6 days from 219 ± 6 in 2000s to 213 ± 7 in 2010s (**Figures 5B, D**). In the western plateau, however, the climate shifts as a pattern of “stable peak precipitation – advancing peak temperature”. The date of peak precipitation changed by 1 day from 207 ± 7 in 2000s to 208 ± 9 in 2010s, and the date of peak temperature advanced by 5 days from 203 ± 6 in 2000s to 198 ± 11 in 2010s. The date of peak growth changes slightly from 225 ± 6 in 2000s to 226 ± 7 in 2010s (**Figures 5A, C**). As a consequence, *δ_G,Pr_* decreased slightly by 1 day, while *δ_G,T_* had a large increase of 8 days between 2000s and 2010s. To sum up, 10% of the grasslands over the TP turned from thermal-constrained ecosystem in 2000s into transition state from thermal- to water- constrained in 2010s, and most of these transitions occur in the semi-humid east (**Figures 5E, F**). Similar transition was also found when using different vegetation indices and GPP products to detect peak growth date (**Supplementary Figure 2**). These shifts in the relative positioning between peak vegetation growth and climate peaks suggest that the alpine grassland ecosystem over the TP is moving towards the tipping point of a transition from thermal- to water-constrained ecosystem.

### 3.3 Impact of shifting peak vegetation growth on ecosystem production

**Figure 6:**
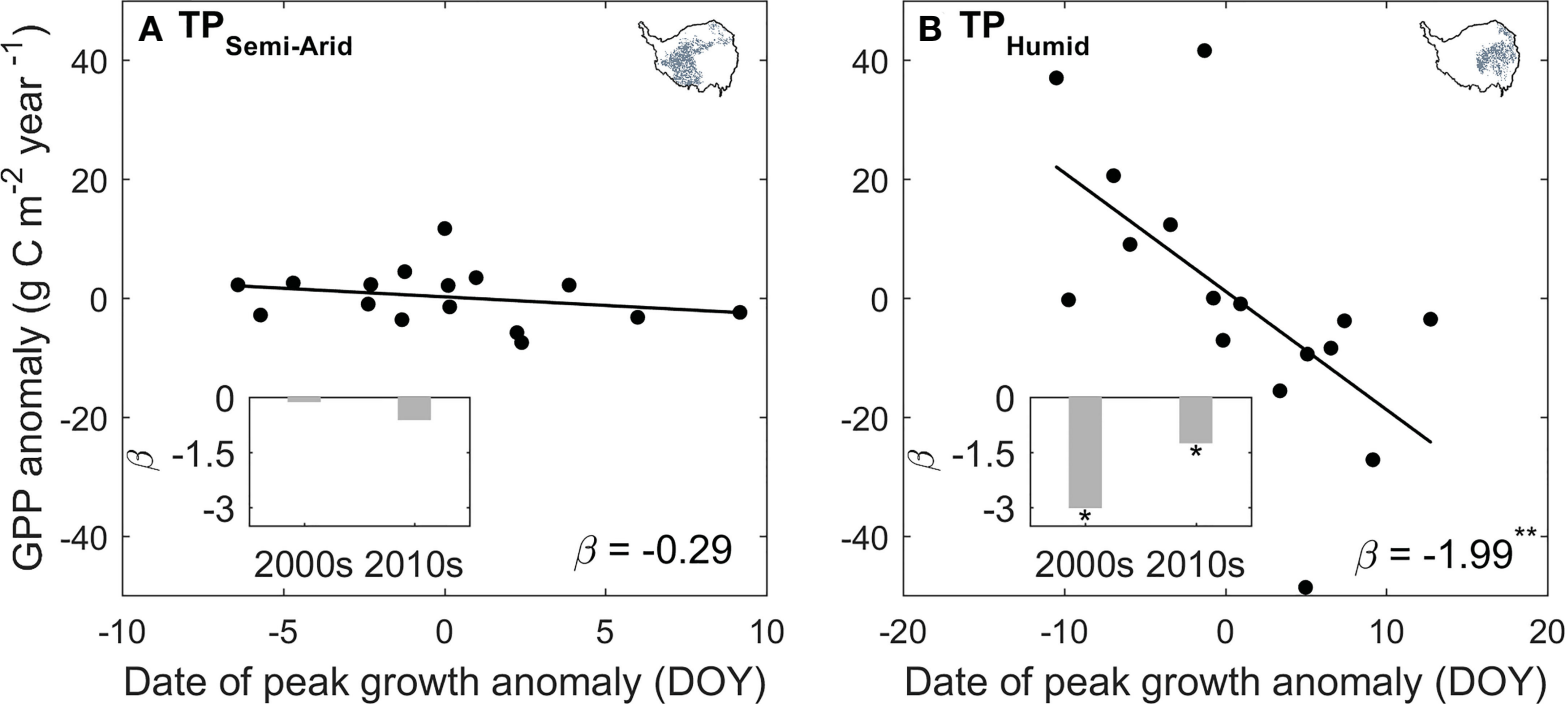
The sensitivity of GPP on the changes in the date of peak vegetation growth for the semi-arid western **(A)** and semi-humid eastern TP **(B)**. The *ß* marks the value of the sensitivity, and the marker * and ** indicates statistically significant at *P* < 0.1 and *P* < 0.05 level, respectively. The inset bar plots show the changes in *ß* between the period 2000s (2001–2010) and 2010s (2011–2020).

We found a notable “earlier peak – larger production” changes for alpine grasslands over the TP. In the semi-humid eastern TP, the ecosystem production increases by 1.99 gCm^-2^ year^-1^ per one day advances in peak vegetation growth, and this sensitivity decreases to 0.29 gCm^-2^ year^-1^day^-1^ in the semi-arid west. The influence of the shifting peak vegetation growth on ecosystem production also changes over time. In the eastern plateau, the sensitivity of ecosystem production to shifting peak vegetation growth (*β*) decreases from -3.02 gCm^-2^ year^-1^day^-1^ during 2000s to -1.25 gCm^-2^ year^-1^day^-1^ during 2010s. In the western plateau, the *β* varies from -0.13 gCm^-2^ year^-1^day^-1^ to -0.62 gCm^-2^ year^-1^day^-1^ for the last two decades and is not significant for both periods (**Figure 6**). This result is robust to the used of GPP dataset (**Supplementary Figure 3**).

## 4 Discussion

We observed an advancing peak vegetation growth over the eastern TP, and a delaying peak vegetation growth over the western TP. This shifting pattern of the peak vegetation growth disagrees with the recent reported delaying peak growth date over the entire TP during 2000–2016 (Wang and Wu, 2019; Yang et al., 2019). It may be caused by the different source of the NDVI dataset. The NDVI data from collection 6 (C6) of the MODIS data used in this study avoided the artefacts of sensor degradation and data processing in GIMMS or collection 5 (C5) of MODIS datasets (Liu et al., 2012; Lyapustin et al., 2014; Zhang et al., 2017).

The advancing peak vegetation growth in the eastern plateau and the delaying peak vegetation growth in the western plateau keeps in pace with the shifts of the onset of start of growing season (Shen et al., 2022), suggesting an earlier (delayed) life cycle of the vegetation growth in the eastern (western) plateau. For carbon cycle, a spring-ward shift of peak vegetation growth means that the period of leaf development and rapid vegetation growth moves toward the summer solstice, when grasses could receive more solar radiation through longer day length (Gonsamo et al., 2018). Thus, advances or delays in vegetation phenology could benefit or hinder the ecosystem production, with increase or decrease in annual GPP for 1.99gCm^-2^year^-1^ day^-1^ and 0.29gCm^-2^year^-1^ day^-1^ in the semi-humid eastern and semi-arid western plateau during 2001–2020, respectively (**Figure 6**). The lower sensitivity of vegetation growth to the shift of peak vegetation growth in the western plateau is induced by the limitation of water availability. In a water-constrained ecosystem, increasing vegetation growth in summer would promote the water demand, thus exacerbate the water stress, and lead to a slow increase or even decrease in vegetation growth (Liu et al., 2018; Hong et al., 2022). The differences of the sensitivity of ecosystem production and peak vegetation growth along the aridity gradient demonstrated that our analyzing framework could well explain the primary climate constrains for the vegetation growth.

**Figure 7:**
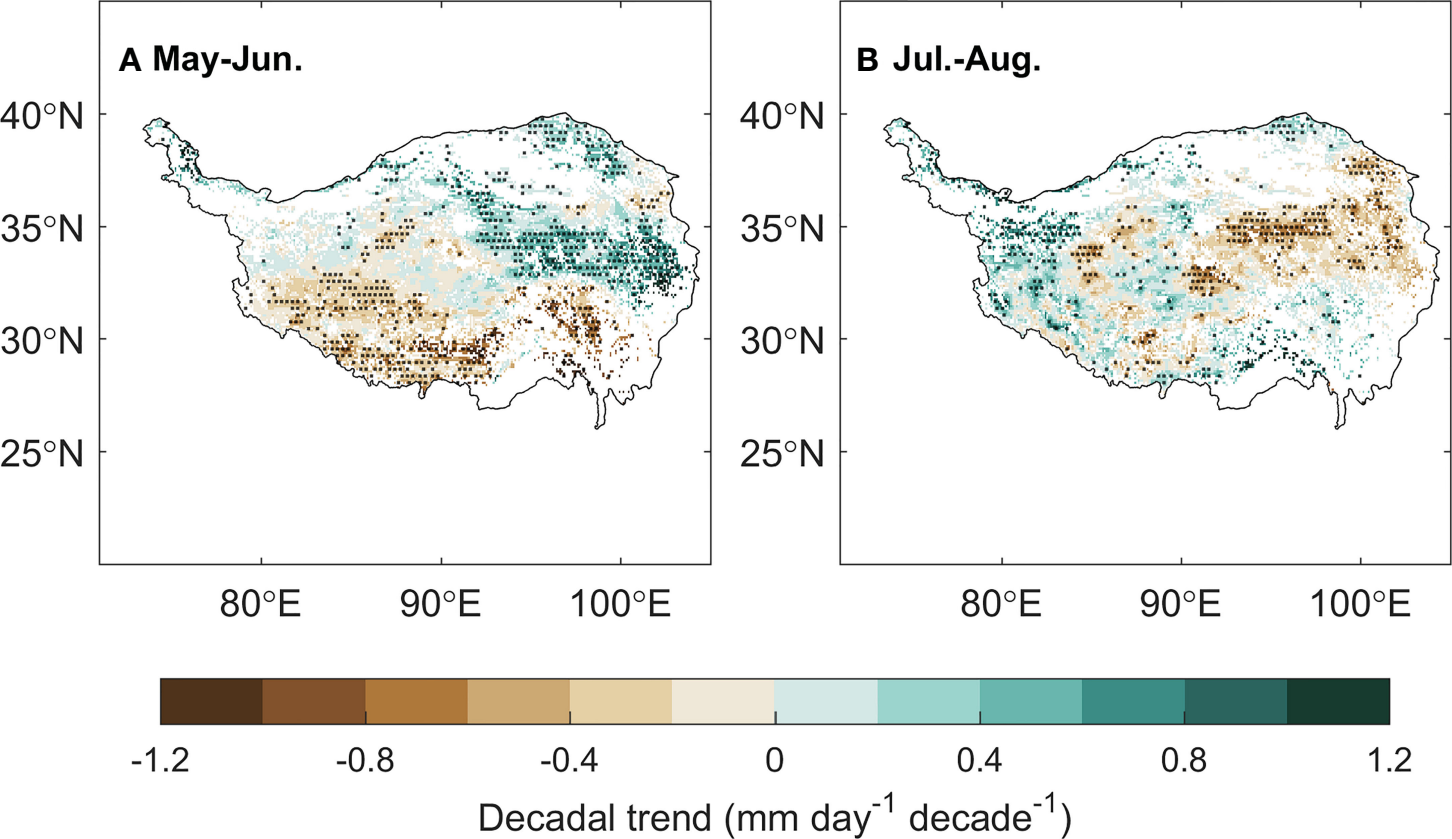
The spatial distribution of the decadal trend of the precipitation in May–June **(A)** and July–August **(B)**.

**Figure 8:**
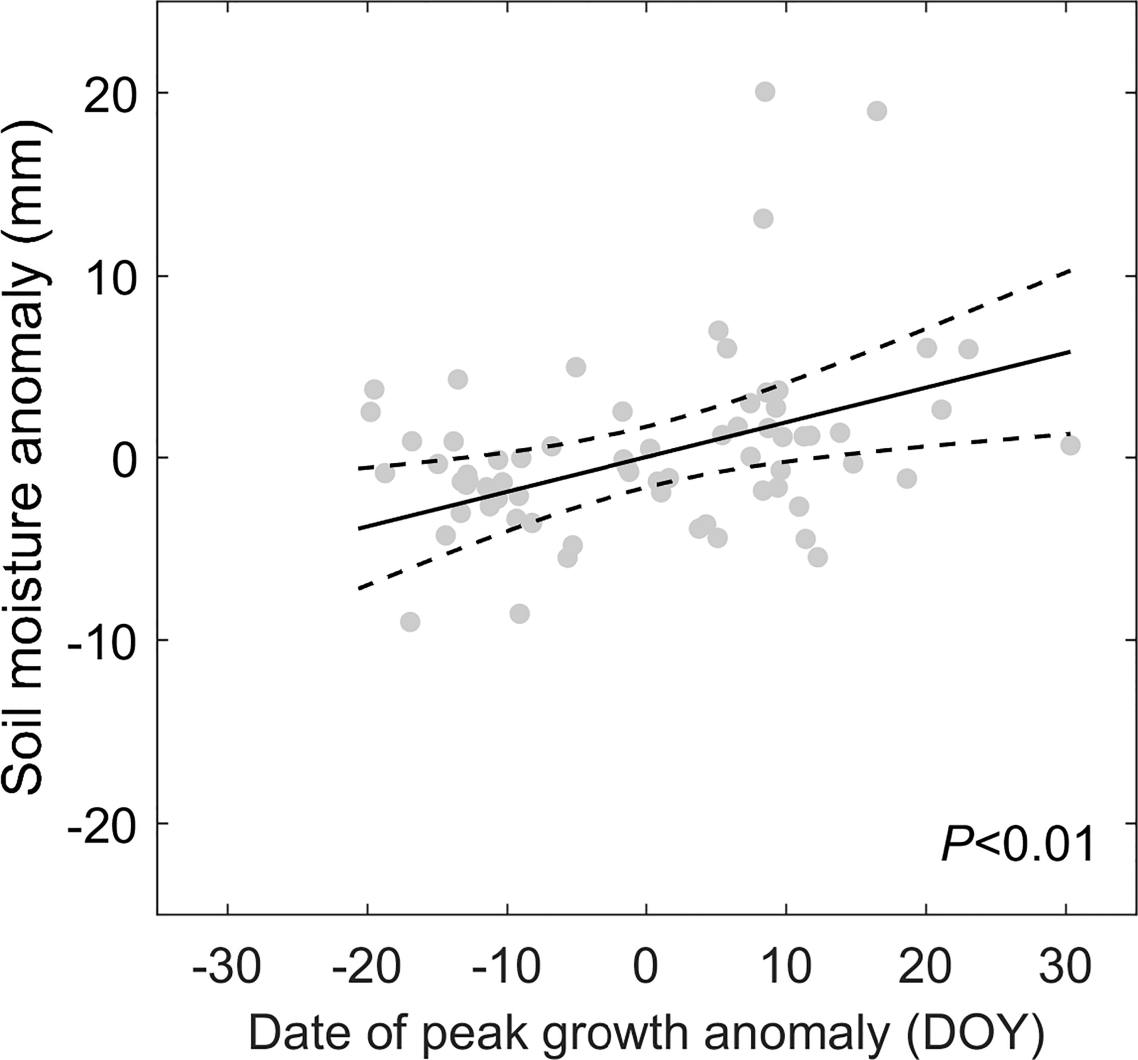
The partial regression plot for the relationship between the date of peak vegetation growth and soil moisture while controlling precipitation. The black line shows the regression line and the dash lines denote the 95% confidence interval.

Applying the framework of “law of minimum” to temporal change of peak vegetation growth provided insights for the changes in the primary climate constrains for the alpine grassland ecosystem over the TP. We found that 2% of the semi-arid ecosystem and 26% of semi-humid ecosystem over the TP is shifting from thermal-constrained ecosystem in to a transition state from thermal- to water-constrained ecosystem (**Figures 5E, F**). This seems to be contradicted to the previously reported increasing precipitation and wetting climate over the TP (Yao et al., 2012; Kuang and Jiao, 2016). We then analyzed the seasonal regime of the precipitation. The increasing precipitation over the last two decades mainly occur during May to June. In July and August, a period when the vegetation growth reaches the peak with largest demand for water, the precipitation significantly decreased at 15% of the region (**Figure 7**). This indicates that changes in precipitation regime only results in blooms in vegetation growth in spring (Shen et al., 2022), instead of a persistent water supply for peak vegetation growth in summer. Moreover, the spring-ward shift of vegetation growth may in turn accelerate the water loss through enhanced evapotranspiration, and lead to more severe water shortage during summer (Buermann et al., 2018). To test this possible mechanism, we further analyzed the relationship between the date of peak growth and the summer soil moisture while controlling precipitation changes. We found a significant positive correlation between the date of peak growth and summer soil moisture (*R* = 0.39, *P* < 0.01, **Figure 8**), which demonstrated that earlier peak growth date could lead to lower soil moisture in summer. This complex interaction of warming and water availability for vegetation growth leads to a weakened relationship between the variation of peak growth date and the annual ecosystem production. We found that the sensitivity of the ecosystem production to advancing peak growth date decreased from -3.02 gCm^-2^year^-1^day^-1^ during 2000s to -1.25 gCm^-2^year^-1^day^-1^ during 2010s. (**Figure 6B**). These results provided additional evidence that the grassland over the eastern TP is shifting from thermal-constrained towards water-constrained ecosystem during the last two decades (**Figures 5E, F**).

In this study, we used precipitation as a proxy of water availability for vegetation growth over the TP, because the precipitation brought by the summer monsoon is the major water input for the water cycle over the TP (Zhang et al., 2017). We did not include the hydrological impact of permafrost thaw in our analyzing framework. There is nonnegligible amount of ice stored in the permafrost over the TP, and melting of the below-ground ice are expected to be an important water source for vegetation growth (Zhao and Sheng, 2019). Nevertheless, the hydrological impact of permafrost thaw is highly variable across space, and it depends on local factors such as terrain, soil properties etc. Thawing permafrost could deepen the impermeable permafrost table and results in drying top soil in well drained regions. In contrast, in poor drained regions and lowlands underlain by continuous and thick permafrost, the top soil generally get wetting after permafrost thaw (Jin et al., 2022). Our understanding of the hydrological impact of permafrost thaw is still rather limited, and we could not answer if the warming induced permafrost thaw would benefit or dampen the vegetation growth over the TP (Jin et al., 2021). Further studies are needed to investigate the complex interaction between permafrost changes and vegetation growth over the alpine grasslands.

## 5 Conclusions

In this study, we used a new framework that based on the law of minimum to analyze the spatial-temporal change of the date of peak vegetation growth of alpine grasslands over the TP. Under this new framework, the timing of peak vegetation growth could act as a proxy for plant’s adaptive state to climatic constrains on its growth, and the shift of its relative positioning against the timing of climate peaks reveal the changes of climatic constrains over the past twenty years. We found an advancing date of peak growth in the semi-humid eastern alpine meadows. Further analysis suggests that this spring-ward shifting peak growth date is driven by the shifting regime of peak precipitation instead of increasing temperature. Our results indicate that the alpine grasslands on the TP are potentially shifting towards a water-limited ecosystem, and highlight the risk of summer drought in a warming climate.

## Data availability statement

The datasets presented in this study can be found in the following links. The MODIS NDVI dataset was derived from https://lpdaac.usgs.gov/products/mcd43c4v061/ and https://lpdaac.usgs.gov/products/mod09a1v061/. The CSIF dataset was derived from https://osf.io/8xqy6/. The GOSIF-GPP dataset was derived from http://data.globalecology.unh.edu/data/GOSIF-GPP_v2/. The PML-V2 GPP dataset was derived from https://data.tpdc.ac.cn/en/disallow/40f57c67-33a6-402d-bd37-6ede91919f23/. The IMDAA climate datasets were obtained https://rds.ncmrwf.gov.in/. The in situ soil moisture observations were derived from https://data.tpdc.ac.cn/zh-hans/data/789e838e-16ac-4539-bb7e-906217305a1d/.

## Author contributions

DL and TW designed the study. DL drafted the manuscript. CX performed the analysis and prepared the figures. All the coauthors contributed to the interpretations of the results and to the text. All authors contributed to the article and approved the submitted version.
