# Supplementary material for: Shifting from a thermal-constrained to water-constrained ecosystem over the Tibetan Plateau
Source: Front Plant Sci. 2023 Apr 19;14:1125288. doi: 10.3389/fpls.2023.1125288 (PMC10154622; doi:10.3389/fpls.2023.1125288)
Supplement: Supplementary file 1 [file DataSheet_1.docx]

## Supplementary Material


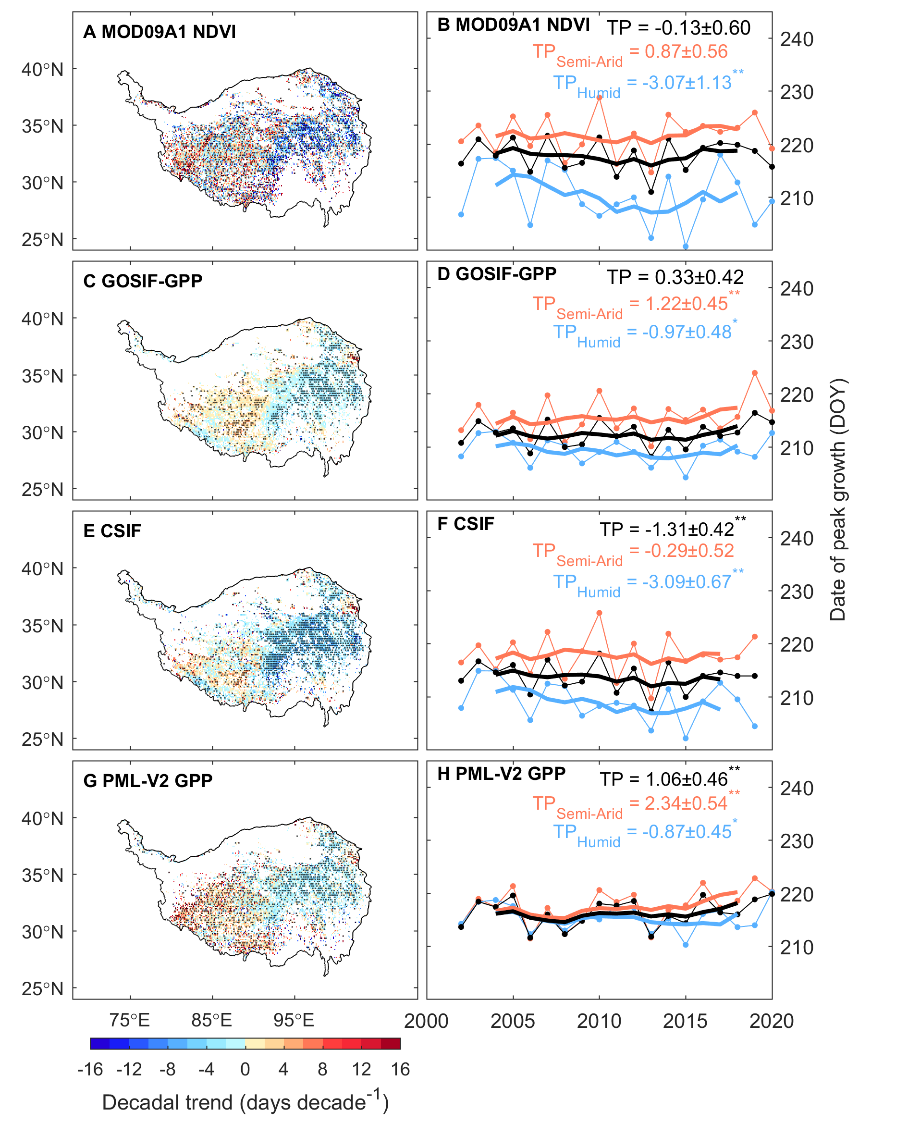


**Supplementary Figure 1.** The temporal trend of the date of peak vegetation growth for the period 2001–2020 estimated from MOD09A1 NDVI, GOSIF-GPP, CSIF and PML-V2 GPP. (**A**, **C**, **E** and **G**) shows the spatial pattern of the trend of the date of peak vegetation growth, and (**B**, **D**, **F** and **H**) shows the time series of the date of peak vegetation growth for the TP (black), semi-arid western TP (orange) and semi-humid eastern TP (blue). Values marked with ** and * suggest that the trend is significant at the level of *P* < 0.05 and *P* < 0.1, respectively. Black dots in the spatial pattern suggest that the trend is significant at the level of *P* < 0.05.


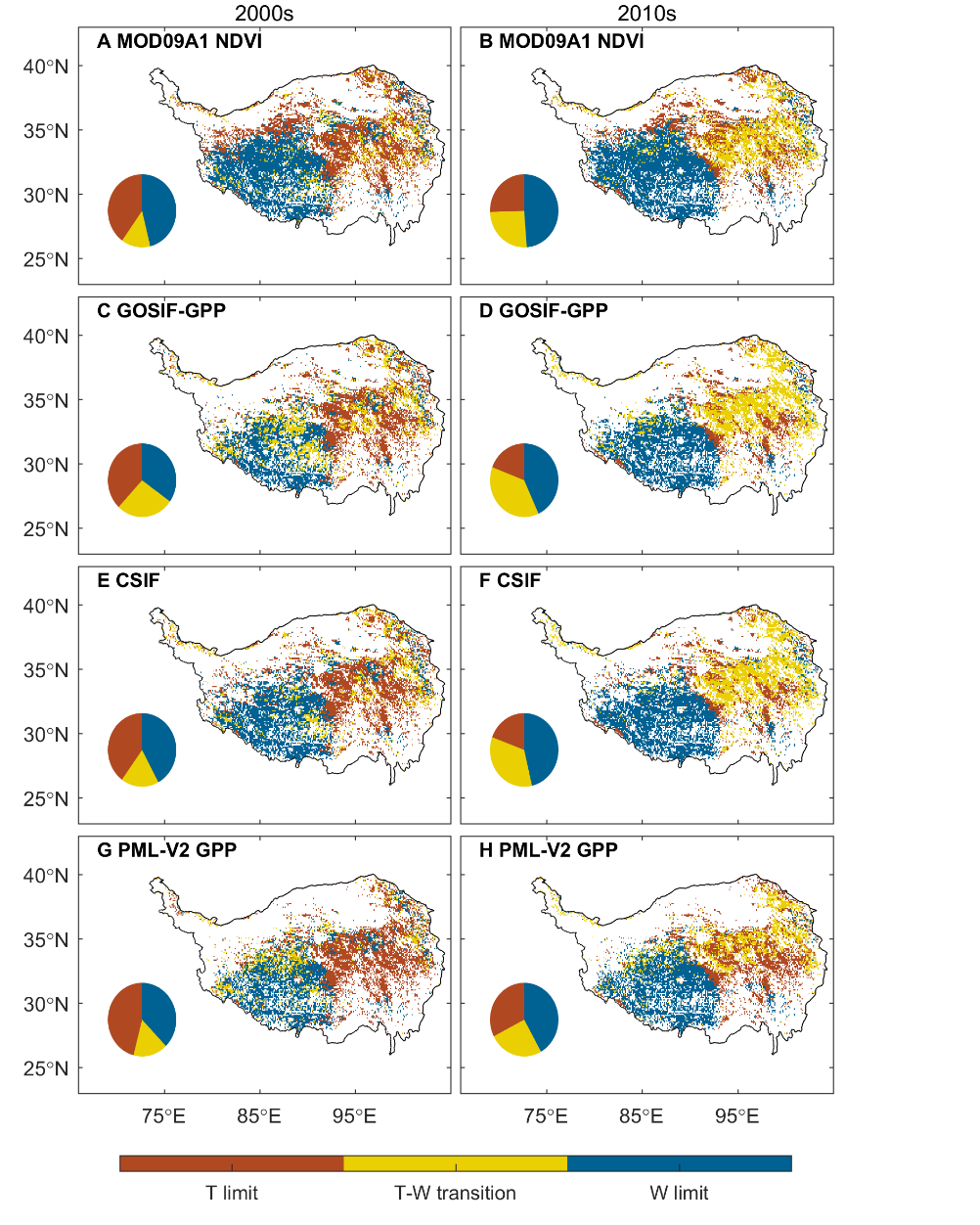


**Supplementary Figure 2.** Spatial distribution of the climate constraints over the TP for the two periods estimated from MOD09A1 NDVI (**A**–**B**), GOSIF-GPP (**C**–**D**), CSIF (**E**–**F**) and PML-V2 GPP (**G**–**H**). The pie plots show the percentage of the regions that are thermal-limited (red), water-limited (blue) or under transition state between thermal- and water-limited ecosystem (yellow).


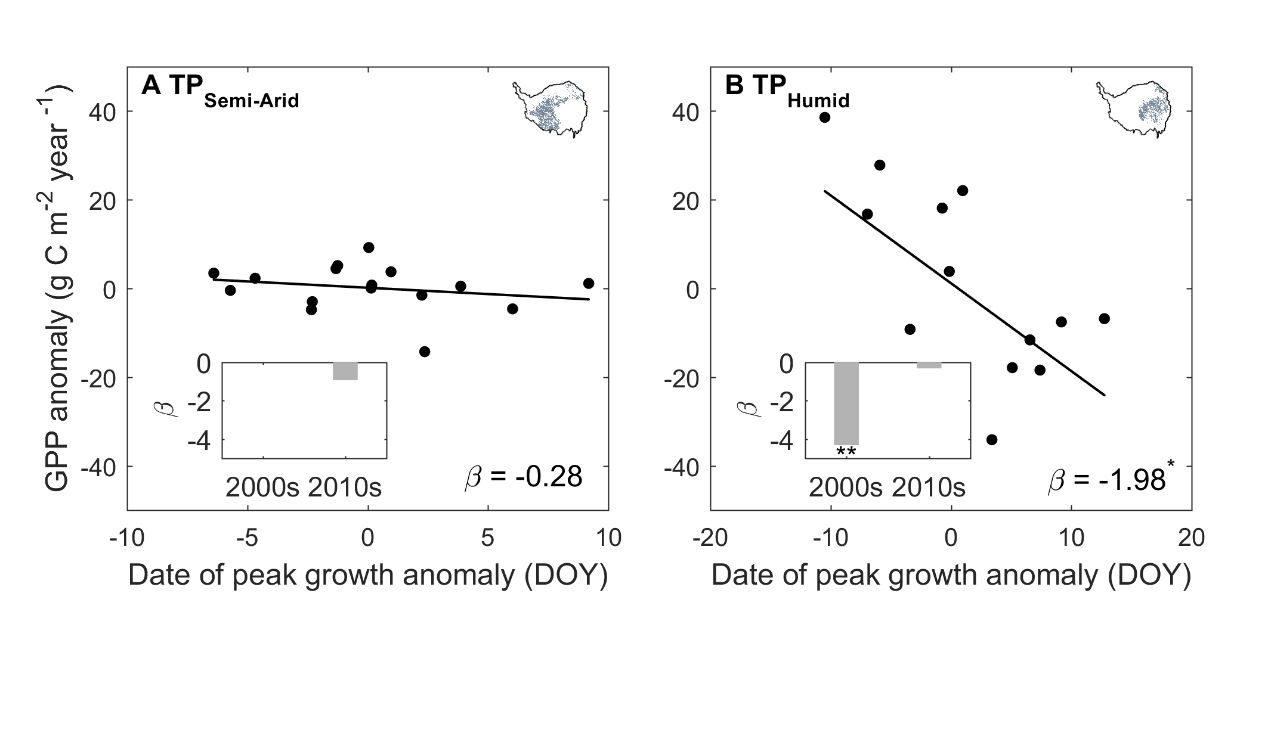


**Supplementary Figure 3.** The sensitivity of GOSIF-GPP on the changes in the date of peak vegetation growth for the semi-arid western (**A**) and semi-humid eastern TP (**B**). The *ß* marks the value of the sensitivity, and the marker * and ** indicates statistically significant at *P* < 0.1 and *P* < 0.05 level, respectively. The inset bar plots show the changes in *ß* between the period 2000s (2001–2010) and 2010s (2011–2020).
